# Reactive Oxygen Species in Embryo Development: Sources, Impacts, and Implications for In Vitro Culture Systems

**DOI:** 10.3390/life16010136

**Published:** 2026-01-15

**Authors:** Sajuna Sunuwar, Yun Seok Heo

**Affiliations:** 1 Department of Biomedical Engineering, Keimyung University, Daegu 42601, Republic of Korea; sazuna13@gmail.com; 2 Department of Mechanical & System Design Engineering, Hongik University, Seoul 04066, Republic of Korea

**Keywords:** reactive oxygen species, oxidative stress, embryo development, IVF, ART, in vitro culture

## Abstract

Reactive oxygen species (ROS) are essential regulators of fertilization and early embryo development in mammals, including humans and various animal models, but they exert detrimental effects when produced in excess. In assisted reproductive technologies (ART), particularly in vitro fertilization (IVF), exposure to non-physiological conditions increases oxidative stress (OS), impairing gamete quality, embryo viability, and clinical outcomes. This review synthesizes experimental and clinical studies describing the endogenous and exogenous sources of ROS relevant to embryo development in IVF. Endogenous ROS arise from intrinsic metabolic pathways such as oxidative phosphorylation, NADPH oxidase, and xanthine oxidase. Exogenous sources include suboptimal laboratory conditions characterized by factors such as high oxygen tension, temperature shifts, pH instability, light exposure, media composition, osmolarity, and cryopreservation procedures. Elevated ROS disrupt oocyte fertilization, embryonic cleavage, compaction, blastocyst formation, and implantation by inducing DNA fragmentation, lipid peroxidation, mitochondrial dysfunction, and apoptosis. In addition, the review highlights how parental health factors establish the initial redox status of gametes, which influences subsequent embryo development in vitro. While antioxidant supplementation and optimized culture conditions can mitigate oxidative injury, the precise optimal redox environment remains a subject of ongoing research. This review emphasizes that future research should focus on defining specific redox thresholds and developing reliable, non-invasive indicators of embryo oxidative status to improve the success rates of ART.

## 1. Introduction

Infertility is becoming an increasingly prevalent global health concern, with both male and female factors contributing to its rising incidence. This growing challenge in human reproduction has driven the rapid expansion of assisted reproductive technologies (ART), particularly in vitro fertilization (IVF). Although IVF is widely used to treat infertility, its success rate remains relatively low while the financial burden on patients remains high. Embryo quality is a critical determinant of IVF outcomes, and multiple variables influence developmental competence, including oocyte and sperm quality, parental health status, time spent manipulating gametes and embryos outside the body, and physicochemical conditions such as temperature, light exposure, and culture media composition. Among these factors, reactive oxygen species (ROS) play a particularly significant role in modulating IVF outcomes. ROS are generated by mammalian oocytes and sperm during fertilization, as well as developing embryos, including zygotes and blastocysts as byproducts of adenosine triphosphate (ATP) production [1]. At physiological concentrations, ROS serve as essential signaling molecules that regulate sperm capacitation, the acrosome reaction, oocyte activation, fertilization, pronuclear formation, implantation, and early pregnancy [2,3,4]. They also contribute to cellular proliferation, cleavage divisions, compaction, blastocyst formation [5,6], and blastocyst hatching [7,8]. Although physiological ROS are indispensable for redox signaling, cellular function and supporting embryo development [9,10], excessive ROS can overwhelm antioxidant defenses, leading to oxidative stress (OS) that disrupts the cellular milieu and impairs fertilization [11,12].

ROS are considered important mediators of oxygen toxicity because of their high chemical reactivity [9]. Different ROS possess distinct biological properties, such as reactivity, half-life, and lipid solubility [13]. For example, the hydroxyl radical (HO^•^) reacts indiscriminately with biological molecules, whereas superoxide (O_2_^•–^) and hydrogen peroxide (H_2_O_2_) exhibit more selective reactivity profiles [9]. Consequently, the various ROS found in embryos have different biological targets and reactivity spectra [6].

Excess ROS generation during mammalian embryonic development can alter gene expression [14], induce lipid peroxidation, and cause DNA damage [14,15]. In mouse zygotes, mitochondrial alterations are often among the earliest detectable signs of oxidative injury [16]. ROS accumulation disrupts mitochondrial structure and function, reduces membrane potential, and interferes with ATP synthesis, ultimately arresting development in mouse and bovine embryos [16,17,18]. Even mild OS can decrease mitochondrial membrane potential (MMP) and initiate pathways leading to cell cycle arrest and programmed cell death in mouse oocytes and zygotes [16,17,19].

**Figure 1:**
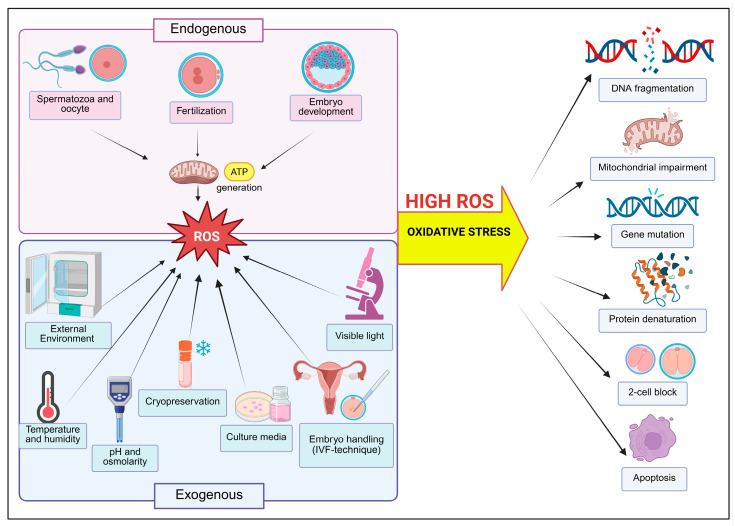
Schematic representation of endogenous and exogenous sources of ROS in the IVF laboratory and their subsequent impact on embryonic development.

Various factors in the IVF laboratory environment, including light exposure, culture media composition, gas atmosphere, cryopreservation methods, and handling techniques, can generate ROS that negatively affect human and animal gametes and preimplantation embryos during in vitro procedures [20,21]. During in vitro handling, these cells may be exposed to excessive ROS originating from both endogenous and exogenous sources (Figure 1). Endogenously, mammalian oocytes and developing bovine and mouse embryos generate ROS through their metabolic activity [22,23]. Exogenous factors that trigger ROS production during in vitro culture include suboptimal culture systems in bovine models [24], high light intensity in mouse zygotes [25], and inappropriate gas atmosphere in bovine and porcine embryos [14,26]. These conditions can damage DNA, arrest embryonic development at the cleavage stage, and induce apoptosis in blastocysts [14]. In vivo, antioxidant systems preserve redox homeostasis, with ROS scavengers present in reproductive fluids; however, preimplantation embryos cultured in vitro lack comparable protection and are therefore more vulnerable to OS.

Elevated ROS levels have been reported to impair embryonic development in multiple species, including human reproductive cells [27], mouse zygote [25], bovine oocytes and embryos [24,28], porcine embryos [29], and ovine embryos [30]. Culture conditions that increase ROS or reactive nitrogen species (RNS) can reduce fertilization rates, impair preimplantation blastocyst quality, and increase cell fragmentation in human and animal models [31]. Mammalian embryos cultured in vitro generate more ROS/RNS than those developing in vivo [20], resulting in reduced fertility, poor developmental outcomes, and lower pregnancy rates following uterine transfer in human clinical IVF and animals models [22,32]. Despite continuous efforts to optimize human and animal embryo culture media and define metabolic requirements for implantation competence, IVF success rates remain suboptimal.

Successful IVF and ART depend on the generation of high-quality embryos, a process that is strongly influenced by both laboratory conditions and maternal and paternal health [33]. Therefore, alongside continued improvements in IVF protocols, monitoring and regulating ROS levels are critical for enhancing oocyte and preimplantation embryo quality and improving IVF outcomes. Accordingly, minimizing OS during ART procedures remains essential across all species, including humans, mice, and bovine models. To address these challenges, this review examines the sources of ROS in IVF and their impact on oocyte maturation and early embryonic development.

## 2. Reactive Oxygen Species (ROS)

ROS have the capacity to generate free radicals and are broadly classified into two categories: radical and non-radical species [34] (Table 1). Free radicals are atoms or molecules containing an unpaired electron in their outer shell, which makes them highly reactive and unstable. As a result, they readily accept electrons from surrounding molecules [35,36]. Owing to their high reactivity, free radicals have a short half-life and often remain localized near their site of production unless rapidly reduced. ROS are oxygen-containing molecules produced during the incomplete reduction of molecular oxygen within cells [37].

**Figure 2:**
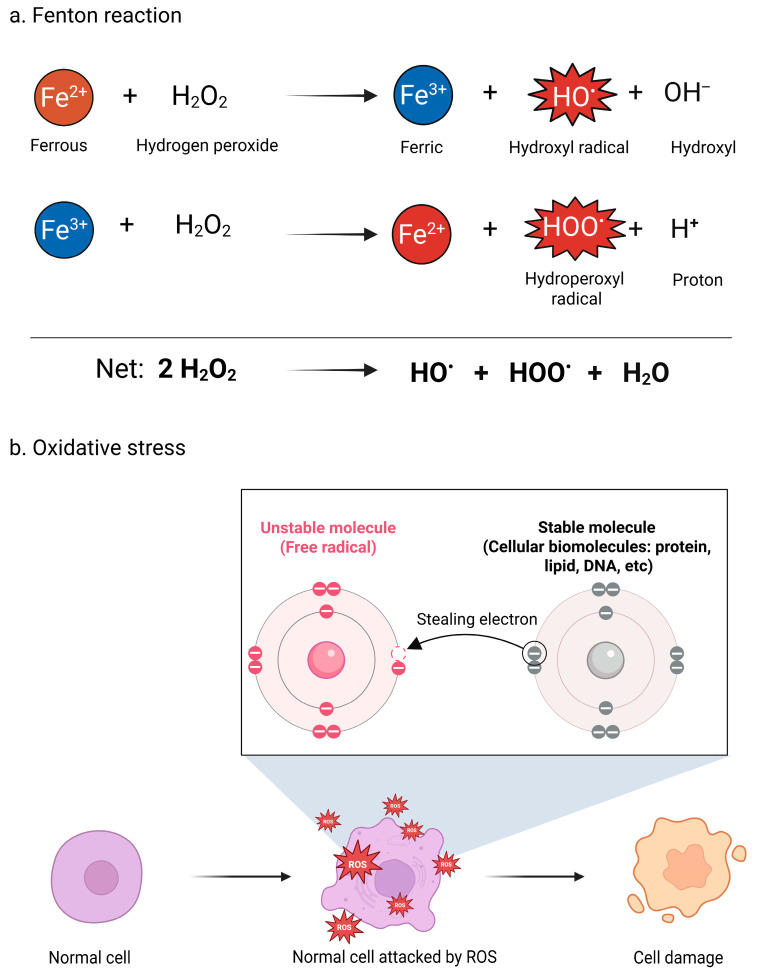
Chemical pathways of ROS generation and cellular damage. (**a**) The iron-catalyzed Fenton reaction. (**b**) Schematic of electron abstraction from biomolecules leading to OS and cell damage.

Radical ROS include O_2_^•–^, HO^•^, peroxyl (RO_2_^•^), hydroperoxyl (HO_2_^•^), and alkoxy radicals (RO^•^) [38]. In contrast, non-radical ROS are generally less reactive because they lack an unpaired electron, which allows them to persist longer and diffuse away from their site of origin, crossing membranes and affecting distant intracellular locations [39]. Examples include H_2_O_2_, hydroxide ion (OH^–^), and organic peroxides (ROOH) [38]. Although less reactive, non-radical ROS can still participate in redox reactions that generate free radicals. For instance, in the Fenton reaction (Figure 2), H_2_O_2_ reacts with reduced transition metals such as ferrous iron (Fe^2+^) or cuprous copper (Cu^+^) to produce the HO^•^, one of the most potent oxidants [40,41]. Similarly, the Haber–Weiss reaction generates HO^•^ through the iron-catalyzed interaction of O_2_^•–^ with H_2_O_2_ [41].

The intracellular movement of H_2_O_2_ is particularly important for maintaining redox homeostasis, as its diffusion enables redox signaling across different subcellular compartments and influences the spatial distribution of antioxidants [42,43].

## 3. Sources of ROS In Vitro Fertilization

### 3.1. Endogenous Sources of ROS

ATP is the fundamental energy currency that drives all cellular functions, and the production of ROS is an inherent byproduct of ATP synthesis [44]. Mammalian spermatozoa, immature sperm, oocytes, and preimplantation embryos are all capable of generating ROS intracellularly [1]. During the various developmental stages of goat preimplantation embryos, intracellular ROS levels fluctuate in accordance with the changing metabolic demands of the developing cleavage-stage embryo and blastocyst [45]. Multiple metabolic pathways and enzymes contribute to endogenous ROS formation in these early cells, including oxidative phosphorylation (OXPHOS), nicotinamide adenine dinucleotide phosphate (NADPH) oxidase, and xanthine oxidase [1]. Among these pathways, mitochondrial OXPHOS, which is directly coupled to ATP generation, represents the primary source of endogenous ROS in mammalian oocytes and embryos.

#### 3.1.1. Oxidative Phosphorylation (OXPHOS)

OXPHOS is the mitochondrial process that generates ATP by coupling oxygen reduction to the formation of high-energy phosphate bonds. It involves a series of redox reactions in which electrons from NADH and FADH_2_ are transferred to oxygen through multiple protein complexes located in the inner mitochondrial membrane, collectively known as the electron transport chain [46]. During this process, molecular oxygen functions as the terminal electron acceptor.

OXPHOS is a defining feature of aerobic respiration, and because developing mammalian embryos have a high and increasing demand for ATP, aerobic respiration plays a central role in supporting early embryogenesis [47]. However, ROS generation is an unavoidable consequence of aerobic metabolism, and excessive ROS can activate signaling pathways that negatively affect oocyte maturation, preimplantation embryo development, and fetal growth across species, including human and rodent models [1,48].

ATP production through OXPHOS is essential for preimplantation embryo development [49,50,51], as it fuels zygotic genome activation and supports subsequent embryonic development [52]. In bovine embryos approximately 90% of ATP is derived from mitochondrial OXPHOS during the pre-compaction stage [53]. In mouse embryos, mitochondrial ATP production accounts for 60–70% of oxygen consumption by the blastocyst stage, compared with only about 30% at the cleavage stage [54]. Interestingly, transient inhibition of OXPHOS during the compact morula stage in vitro has been reported to enhance developmental outcomes in both porcine [55] and bovine embryos [49]. This effect is attributed to partial suppression of mitochondrial ATP synthesis during the peri-compaction period, which balances glycolysis versus OXPHOS-derived ATP while establishing a favorable intracellular redox state. In contrast, partial inhibition of OXPHOS from the one-cell zygote stage has been shown to exert detrimental effects on porcine embryo development [55], supporting the notion that OXPHOS is essential during early embryonic development prior to the later shift toward increased glycolysis.

#### 3.1.2. Nicotinamide Adenine Dinucleotide Phosphate Oxidase (NADPH Oxidase)

NADPH oxidase is a membrane-bound enzyme complex that catalyzes the oxidation of NADPH to NADP^+^. This reaction drives the reduction of molecular oxygen, resulting in the formation of O_2_^•–^ in mouse and other mammalian cell models [7,56]. Unlike mitochondrial ROS, which arise as an unintended byproduct of respiration, NOX-derived ROS are produced deliberately, primarily in the form of H_2_O_2_ and O_2_^•–^, as observed in various mammalian species [57,58]. NOX activity has been reported on the surface of rabbit blastocyst-stage cells, where it generates H_2_O_2_ and O_2_^•–^ through electron transfer from NADPH to oxygen [59]. In sea urchin oocytes, NOX also participates in ROS generation during fertilization, particularly in the production of H_2_O_2_ [60]. Diphenyleneiodonium (DPI) is a well-established NOX inhibitor. In mouse 2-cell stage embryos, DPI treatment reduces H_2_O_2_ production in a concentration-dependent manner; however, prolonged exposure to DPI has been reported to have detrimental effects on embryonic development [7].

#### 3.1.3. Xanthine Oxidase

Xanthine oxidase contributes to ROS generation in mammalian embryos as a byproduct of purine metabolism [61,62]. In mouse embryos, hypoxanthine induces toxicity by producing O_2_^•–^ during its conversion to xanthine and subsequently to uric acid through the action of xanthine oxidase, particularly at the 2-cell stage [7]. Under in vitro culture conditions, purines have been shown to impair preimplantation mouse embryo development [1,63], with hypoxanthine specifically reported to induce a 2-cell stage developmental arrest [64].

Supporting the involvement of xanthine oxidase-derived ROS, the use of xanthine oxidase inhibitors, such as allopurinol and oxypurinol, has been shown to reduce peroxide levels without adversely affecting cleavage-stage morphology in bovine embryos [65], with similar protective effects observed in mouse embryos [7]. During preimplantation development in mice, xanthine has been suggested to represent the final product of purine metabolism [61]. However, mouse blastocysts do not exhibit measurable xanthine oxidase activity [66], suggesting that purine-derived ROS production is confined to early cleavage stages. The degradation of purine nucleotides by xanthine oxidase during the mouse 2-cell block may therefore contribute to elevated ROS levels and compromised early embryonic development.

While endogenous metabolic pathways are inevitable sources of ROS, the IVF laboratory environment introduces a variety of external factors that can further exacerbate ROS production.

### 3.2. Exogenous Sources of ROS

#### 3.2.1. Temperature

In vivo, oxygen scavengers present in human follicular fluid such as vitamins A, C, and E, pyruvate, glutathione (GSH), taurine, and cysteamine, help protect embryos from OS [67,68,69]. In ART settings, however, exposure of embryos to ambient temperature is unavoidable during handling, and temperature fluctuation is one of the factors known to alter embryonic metabolism [70]. Altered temperature is a recognized risk factor for inducing metabolic, physiological, cellular, and molecular changes in the female reproductive tract, potentially leading to infertility [71].

With global temperatures steadily rising [72], humans and animals are increasingly exposed to heat stress, particularly during summer months, which can reduce fertility by pushing physiological systems beyond their optimal limits [73]. Numerous studies have reported that elevated temperatures disrupt oocyte maturation in bovine [74], mouse [75], and porcine models [76]. Heat-induced physiological alterations can damage intracellular components through OS, triggering the apoptosis pathway [77]. During oocyte maturation, excessive production of free radicals is triggered by heat stress [78], and elevated temperature has been shown to alter nuclear and cytoskeletal structures, resulting in delayed embryo development [79]. Increased ROS production, combined with reduced antioxidant defense mechanisms, has been associated with the detrimental effects of heat stress on bovine oocytes and embryos [80,81]. Similarly, heat stress has been shown to decrease GSH levels and increase H_2_O_2_ levels in mouse oocytes and zygotes [82].

Despite precise laboratory equipment, the temperature of incubators or heating plates often does not accurately reflect the actual temperature of the culture media. Therefore, relying solely on external incubator displays for temperature accuracy is inappropriate. Human IVF studies have shown that when incubator temperatures are maintained slightly below 37 °C, pregnancy rates are higher compared with temperatures above 37 °C [83], although the optimal temperature for in vitro culture remains a subject of debate [84].

Additional studies demonstrate species-specific sensitivity to heat stress. In buffalo oocytes, incubation under heat stress increases ROS, lipid peroxidation, and nitric oxide levels [85]. In cattle, heat stress elevates free radical production, contributing to meiotic arrest, reduced oocyte quality, and decreased embryo development rates [86,87]. In mice, oocyte cytoplasmic maturation appears more vulnerable to heat stress than nuclear maturation [75], while in pigs, heat stress has been shown to enhance autophagy induction in oocytes [88].

Given these findings, it is essential to consider how handling procedures and external environmental factors influence heat loss from the culture media to maintain the appropriate temperature for human oocytes and embryos during in vitro culture [84]. Careful monitoring and precise temperature control throughout the entire ART process are therefore critical to supporting optimal embryo development and reducing the risk of OS-induced damage.

#### 3.2.2. Light

Light exposure is inevitable in ART laboratories during embryo handling, manipulation, and quality assessment. This represents an environmental factor that is largely absent in vivo. Throughout evolution, embryos developed within the dark uterine environment, and therefore protective mechanisms against light exposure may not have evolved. During ART procedures, oocytes, sperm, and embryos are exposed to light at several steps, including oocyte retrieval, placement and removal of culture dishes from incubators, microscopy for ICSI (intracytoplasmic sperm injection), fertilization checks, morphological assessments, and embryo transfer.

Several variables must be considered when evaluating the effects of light exposure, including duration, intensity, and wavelength. Light can affect cells in multiple ways: it may directly stress cells, activate stress-responsive genes, or cause DNA strand breaks through indirect oxidative damage [89,90]. Light can also exert indirect effects by oxidizing components within the culture media or overlay oil, potentially converting neutral or beneficial substances into toxic compounds. Oxidation of culture oil used in human embryo culture has been linked to light exposure [91].

One of the earliest studies reporting light-induced cellular toxicity was conducted by Hill Jr. et al. [92]. The study found that protozoa placed in an acridine dye solution were killed following light exposure, not due to direct light toxicity but rather due to photochemical changes in acridine orange [92]. When culture media containing HEPES and riboflavin were exposed to light, cell toxicity occurred due to ROS generation in mammalian cells [93,94,95].

Studies have shown that mammalian embryos exhibit stage-dependent sensitivity to light exposure. Rabbit ova exposed to light for up to 12 h displayed delayed cleavage rates, even when filters were used [96]. Similarly, day-1 rabbit zygotes exposed to as little as 1 h of light exhibited reduced proliferation, indicated by decreased thymidine incorporation, suggesting that early-stage zygotes are more sensitive to light than later-stage morula or blastocyst [97]. In contrast, limited exposure of rabbit oocytes and zygotes to 3250 lux of fluorescent light for 30 min did not adversely affect embryonic development or implantation [98].

In addition to developmental stage, wavelength is a critical determinant of light-induced embryotoxicity. Exposure of hamster embryos to short-wavelength visible light, particularly blue light (445–500 nm), increases Hsp70 expression, ROS generation, and blastomere apoptosis compared with longer-wavelength red light (620–750 nm) [99]. Consistent with this, mouse and hamster embryos were exposed to cool white fluorescent light, which contains a high proportion of short-wavelength visible light, generates more ROS than embryos exposed to warm white fluorescent light, leading to a 2-cell block in mouse embryos [25].

Light intensity further modulates embryonic responses as high-intensity light exposure can influence mouse embryos either directly or indirectly by increasing ROS generation, altering gene transcription, or producing degradation products in the culture media [100]. Multiple studies have confirmed that the damaging effects of light increase as wavelength decreases; accordingly, longer-wavelength red light (550–730 nm) is less harmful to human and mouse embryos than shorter-wavelength blue light [101,102].

Supporting this wavelength specificity, a study showed that mouse embryos exposed to yellow light (590 nm) exhibited reduced blastocyst formation rates, whereas embryos exposed to green (520 nm), blue (470 nm), or red (620 nm) light increased the numbers of H2AX-positive cells, an indicator of DNA damage. These alterations were associated with lower pregnancy rates and reduced fetal weights following embryo transfer [103]

Given the sensitivity of early-stage embryos to light-induced injury, minimizing exposure duration and utilizing longer wavelength lights are essential laboratory practices to reduce ROS generation and preserve developmental competence.

#### 3.2.3. Oxygen Concentration

The oxygen concentration in the mammalian oviduct is significantly lower than atmospheric levels [104,105]. When equilibrated with atmospheric oxygen, the oxygen concentration in culture medium reaches approximately 224 µmol/L at 37 °C [106]. Although oxygen settings vary among ART laboratories, human embryos are most commonly cultured at either atmospheric oxygen concentration (~20%) or at the recommended low-oxygen condition of ~5% [107]. In contrast, oxygen levels in most human and mammalian tissues typically range from 2% to 6%, which is three to nine times lower than the ~20% oxygen concentration used in standard cell culture conditions [108].

Mouse embryos cultured at atmospheric oxygen (~20%) exhibit substantial gene deregulation, including genes involved in cell growth and gastrulation [109]. High-oxygen environments have also been associated with altered DNA methylation and reduced blastocyst rates in bovine embryos [109,110]. Reducing oxygen concentration from approximately ~20% to ~5% during in vitro culture improves embryonic development across multiple species [111,112,113,114]. Bovine embryos cultured at atmospheric oxygen display elevated intracellular ROS levels and a higher incidence of irreversible developmental arrest compared with embryos cultured at 5% oxygen [115]. Consistent with this, in both mouse [116] and bovine [117] embryos, ROS production increases under atmospheric oxygen, whereas reducing oxygen concentration enhances development and helps overcome the mouse 2-cell block [118].

Mechanistically, exposure to atmospheric oxygen promotes OS during in vitro culture by increasing the activity of oxygen-dependent oxidase enzymes and overwhelming antioxidant defenses, thereby delaying the mammalian embryo development [32,119,120].

Beyond fixed oxygen concentrations, recent studies have investigated oxygen consumption as an indicator of embryo viability. Electrochemical approaches have been utilized to measure the oxygen consumption rate of individual mice and bovine embryos as a potential marker of developmental competence [121,122,123]. Kuno et al. employed a chip-based embryo respiration monitoring system (CERMs) for preclinical evaluation in a chimeric mouse model. Their findings indicated that oxygen consumption measurements obtained using CERMs did not compromise developmental outcomes following embryo transfer [124]. Notably, embryo size and mitochondrial DNA copy number were found to be independent of the oxygen consumption rate.

Collectively, these findings demonstrate that culturing embryos under physiologically low oxygen tension more closely mimics the in vivo environment, limiting ROS overproduction, and supports optimal embryonic development and viability in vitro.

#### 3.2.4. Culture Media

In ART laboratories, various types of culture media are used, each with different constituents, some of which may contribute to ROS generation [125]. In the IVF laboratory, the culture medium serves as the immediate environment for gametes and embryos, and its specific formulation is a primary determinant of redox balance during in vitro development. Elevated ROS levels in culture media have been associated with human embryo fragmentation and poor blastocyst quality [22,126]. However, other studies have reported no significant association between ROS levels in the culture medium and human embryo quality, blastocyst formation, or developmental arrest [127]. Thus, the precise role of ROS during the earliest stages of embryonic development remains unclear.

Although the use of commercial culture media has improved human embryo quality, varying levels of ROS have still been detected depending on media composition [128]. Supplementation of culture media with antioxidants helps maintain the pro-oxidant-antioxidant balance within mammalian embryos [20].

In addition, culture media volume is a critical determinant of the embryonic microenvironment, as embryo development rates are higher in microdroplet culture systems compared with conventional large-volume systems (1 mL) [129]. Moreover, culture media containing trace amounts of metallic ions such as Zn, Fe, and Cu can generate harmful ROS through Fenton and Haber–Weiss reactions [130,131]. These metallic cations, commonly introduced through water or chemicals used in media preparation, may negatively affect embryonic development. Notably, the addition of metal ion chelators such as EDTA or transferrin reduces excessive ROS production and helps overcome mouse 2-cell developmental block in vitro [129,132,133,134].

To mitigate the risk associated with culture media and external environment-induced OS, research has increasingly focused on the targeted supplementation of culture media with protective agents, including antioxidants as well as other redox modulating and chelating compounds.

##### Use of Antioxidants in Culture Media

Antioxidants are either natural or synthetic compounds. Natural antioxidants are found in fruits, vegetables, and fish and play a crucial role in minimizing OS within the body [135]. Synthetic antioxidants, on the other hand, are derived from natural substances or are fully synthetic [136]. Antioxidants reduce OS by neutralizing ROS, thereby protecting cellular components and genetic material from oxidative damage. Interest in adding antioxidants to embryo culture media has increased in recent years because these media have relatively simple compositions and contain fewer antioxidant molecules than those present in vivo. Some studies suggest that supplementing IVF culture media with antioxidants can counteract ROS production. To reduce the risk of OS and its damaging effects on mammalian gametes and embryos, antioxidants such as isoflavones, urate, ascorbic acid, taurine, hypotaurine, genistein, alpha-lipoic acid, melatonin, and vitamin E have been used in IVF culture systems [137,138,139,140,141].

The use of a combination of antioxidants (acetyl-L-carnitine, N-acetyl-L-cysteine, and alpha-lipoic acid) in IVF mouse embryo culture medium has been shown to accelerate development from the 5-cell stage to the expanded blastocyst stage, increasing total cell number while maintaining intracellular GSH levels [142]. In addition, supplementing IVF media with this antioxidant combination during mouse oocyte and sperm collection improved developmental progression from the 2-cell stage to the expanded blastocyst stage and reduced intracellular H_2_O_2_ levels [143]. Similarly, incorporating these antioxidants into cryopreservation procedures for mouse blastocysts increased post-thaw viability and enhanced developmental potential both in vitro and after uterine transfer by reducing OS [144]. A recent prospective, randomized, multicenter clinical trial also reported that adding acetyl-L-carnitine, N-acetyl-L-cysteine, and alpha-lipoic acid to embryo culture media improved implantation and pregnancy rates in women in their mid- to late 30 s [145].

In addition, melatonin is another antioxidant used in IVF. It is a hormone produced in the pineal gland of vertebrates and is also present in various other tissues and organs, including the uterus, placenta, ovaries, and bone marrow [146,147]. Both mouse and bovine cumulus cells and oocytes have been shown to express melatonin receptors [148,149]. Melatonin has multiple physiological roles: it modulates circadian rhythms, enhances immune function, and regulates reproductive capacity in mammals. It is also a potent free radical scavenger [150,151], acting both by directly neutralizing ROS and by upregulating the expression of several antioxidant genes [152]. Melatonin downregulates pro-apoptotic genes (*Bax* and *caspase-3*) and upregulates the anti-apoptotic gene *Bcl-2*, thereby reducing ROS accumulation [153,154]. Supplementation of melatonin in embryo culture media has produced long-term beneficial effects on embryo development in murine [154,155], bovine [156,157,158], porcine [159,160,161], and ovine species [162]. Melatonin concentrations of 10^−7^ M in culture media have been shown to improve the quality of microinjected pronuclear embryos by reducing OS and apoptosis [163]. Similarly, the addition of compounds such as resveratrol, coenzyme Q10, vitamin C, vitamin E, alpha-lipoic acid, baicalin, apigenin, β-cryptoxanthin, c-phycocyanin, quercetin, retinol, and L-carnitine to embryo culture media has been shown to enhance embryo development, improve blastocyst survival after cryopreservation, and increase blastocyst cell numbers in mammals by reducing intracellular ROS levels [164,165].

Beyond culture media supplementation, OS originating from maternal physiological conditions can also influence embryo development. Maternal health plays a critical role in achieving a successful pregnancy. Alcohol consumption during pregnancy increases ROS levels, which can impair embryonic development. The hepatoprotective effect of betaine against ethanol-induced ROS is well established [166]. Supplementing culture media with 50 µg/mL betaine has been shown to rescue ethanol-damaged mouse embryos by markedly reducing ROS levels [167]. Excessive ROS may also contribute to the developmental abnormalities observed in the offspring of diabetic mothers. Pregnant diabetic rats supplemented with antioxidants such as vitamin E [168], vitamin C [169], or lipoic acid [170] produce offspring with fewer malformations.

Despite these findings, the use of antioxidant supplementation in ART does not always confer additional benefits. Clinical studies in human reproduction have produced conflicting results, and the role of antioxidants remains a subject of considerable debate [171]. Therefore, more comprehensive in vivo and in vitro studies are required before the therapeutic value of antioxidants for infertile men, infertile women, or IVF procedures can be firmly established.

In addition to chemical supplements and antioxidants, the physical character of the culture medium, particularly stable pH and osmolarity, are crucial for maintaining cellular homeostasis and limiting oxidative damage.

##### pH of Culture Media

One of the crucial parameters in embryo culture is maintaining an appropriate external pH (pH_e_) to minimize stress on gametes and embryos and to create an optimal in vitro environment [172]. In bicarbonate-buffered media, pH_e_ is determined by the balance between the CO_2_ concentration in the incubator and the amount of bicarbonate in the medium. The physiological pH of human oocytes and embryos varies slightly during development, generally ranging from 6.98 to 7.03 in oocytes [173] and around 7.12 in embryos [174]. Even small deviations from these ranges can disrupt intracellular processes and negatively influence development. For example, altering the internal pH (pH_i_) of hamster embryos for only three hours, either increasing it to about 7.4 or decreasing it to about 6.8, changes the localization of mitochondria and actin microfilaments compared with controls maintained at about 7.2 [175]. In mouse embryos, developmental competence can also be compromised by modest increases in pH_i_ of 0.1 to 0.15, which substantially enhance embryonic glycolysis and reduce oxidative metabolism [176]. Furthermore, vitrification has been reported to reduce the ability of hamster 2-cell embryos to regulate pH_i_ for approximately six hours after warming [177].

In addition to these intrinsic effects, external factors influencing pH_e_ can themselves act as stressors. Swain, Pool, and coworkers noted that pH_e_ can be influenced by temperature, with certain media exhibiting a rise in pH_e_ as temperature decreases [178]. This indicates that stable pH regulation requires meticulous temperature control during handling and incubation. Supporting this, one-cell mouse embryos maintained at a pH_i_ of 7.1 for 19 h developed significantly fewer blastocyst cells, showed higher levels of apoptosis, and produced smaller and lighter fetuses compared with controls [179].

Compared with embryos, denuded oocytes are unable, or only partially able, to regulate their pH_i_ near 7.1 [180]. For this reason, the pH_e_ of the culture medium must be adjusted to achieve a slightly higher target pH_i_ of 7.2 to 7.3 [180,181]. pH can change rapidly during routine handling; for example, when a culture dish without an oil overlay is removed from the incubator and exposed to an atmospheric, non-gassed environment, the pH begins to rise within one minute [174]. Likewise, fluctuations in temperature or CO_2_ concentration during human and murine embryo incubation markedly alter media pH, leading to impaired development and poorer clinical outcomes through increased OS [84]. Alterations in mitochondrial pH gradients can further contribute to elevated production of ROS [182] and nitric oxide [183]. Thus, alterations in pH within the mitochondrial respiratory chain are considered an additional stimulant of OS [184].

Alongside pH regulation, careful control of culture medium osmolarity is necessary, as osmotic imbalances can further amplify metabolic stress and ROS accumulation.

##### Osmolarity of Culture Media

The osmolarity of embryo culture media is considered a critical factor for the success of ART [185]. It can be influenced by multiple variables, including dish preparation, culture media volume [186], the use of mineral oil [187], culture dish design [188], and the level of humidification in incubators [189]. Media osmolarity, which reflects the osmotic pressure of a solution, plays a key role in mammalian cell volume regulation and preimplantation embryo development [190,191]. Uninterrupted culture systems provide advantages such as reduced handling and improved culture stability; however, prolonged culture inevitably leads to an increase in medium osmolarity over time [192]. Even brief exposure of preimplantation mouse zygotes to high-osmolarity media (>300 mOsm) can impair developmental progression [193]. Conversely, culturing mouse embryos in low-osmolarity medium (250 mOsm), compared with high osmolarity (>300 mOsm), significantly decreases the incidence of the 2-cell block [194].

Human embryo development and clinical outcomes can be substantially affected by hypertonic culture conditions [189]. These adverse effects are attributed to several mechanisms, including OS, cell shrinkage, DNA and mitochondrial damage, cell-cycle arrest, and apoptosis [195]. Additionally, studies have reported that dry incubator conditions may elevate osmolarity and OS, thereby negatively affecting human embryo development [189,195,196]. Nevertheless, the most recent Cochrane review found no clear evidence of a difference [197].

Ultimately, precise control of osmolarity, together with culture media composition is essential to maintain the osmotic balance required for embryo viability. The combined effects of these environmental stressors highlight the need for protective strategies, particularly during high-stress procedure such as cryopreservation, where osmotic disturbances and oxidative challenges are most pronounced and further compromise embryonic redox balance.

## 4. Relationship Between ROS and Cryopreservation

Sperm, oocytes, ovarian tissue, and embryonic tissue are among the various cells and tissues that have been successfully preserved using cryopreservation [198,199,200,201]. The use of cryopreservation in ART programs has been steadily increasing, and the preservation of gametes and embryos is now a fundamental component of modern reproductive medicine. This technology enables individuals to store high-quality oocytes and embryos for future pregnancies or for use in surrogacy programs.

Before the development of vitrification, slow freezing was the most widely used method for cryopreservation of human and mammalian gametes and embryos [202]. However, compared with vitrification, slow freezing has been shown to induce OS, cellular damage, and apoptosis in human oocytes and cleavage-stage embryos due to the formation of intra- and extracellular ice crystals [203]. Several steps in the cryopreservation process can contribute to OS, including exposure to cryoprotectants, fluctuations in pH and osmolarity, cooling and warming processes, and the dilution of sperm samples. High levels of ROS generation during cryopreservation have been documented in human gametes as well as in mice and human embryos [204,205,206].

During oocyte cryopreservation, ROS may originate from both intracellular and extracellular sources [207]. External factors include cryoprotectants such as dimethyl sulfoxide (DMSO), which can trigger the release of large amounts of calcium ions (Ca^2+^) from the endoplasmic reticulum into the cytoplasm. The subsequent uptake of excess Ca^2+^ by mitochondria further enhances ROS production [208]. In addition, the abrupt temperature changes that occur during freezing and thawing contribute to increased ROS formation. For example, heat stress during thawing has been shown to disrupt mitochondrial function and alter Ca^2+^ influx, thereby reducing the in vitro maturation rate of vitrified goat oocytes [209].

Cryopreservation not only elevates ROS generation in oocytes but also damages intracellular organelles such as the endoplasmic reticulum and mitochondria as a consequence of OS [207]. In contrast, vitrification involves ultra-rapid cooling, which minimizes ice crystal formation and reduces osmotic shock, resulting in less cellular damage [210].

Ultimately, the extent of oxidative damage incurred during freezing and thawing cycles represents a pivotal factor influencing post-thaw embryo viability, directly impacting subsequent implantation rates and the overall success of the IVF procedure.

Beyond the artificial environment of the IVF laboratory, the physiological biological fluids that naturally surround the oocyte, sperm, and embryo in vivo also play a critical role in mediating oxidative status.

## 5. Relationship Between ROS and Oocyte, Follicular Fluid, and Tubal Fluid

**Figure 3:**
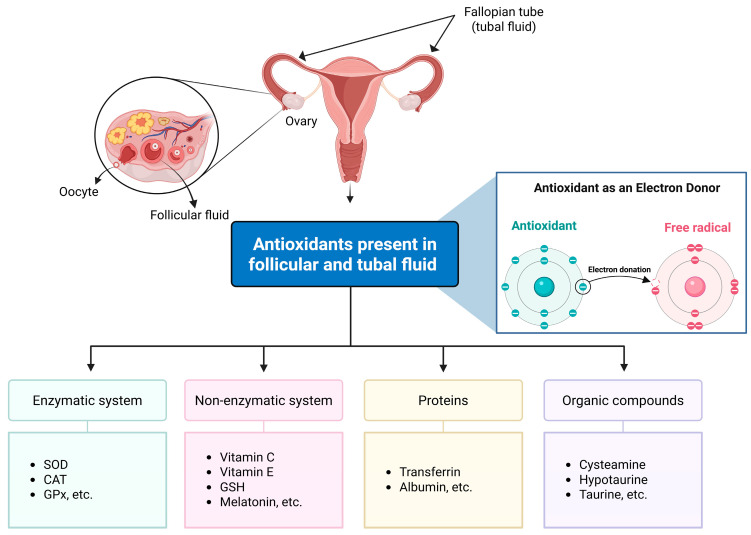
Schematic overview of antioxidant defense systems in follicular and tubal fluids.

In vivo, embryos and their surrounding environments possess multiple defense mechanisms against OS [211]. Follicular fluid (FF) provides the microenvironment essential for oocyte development and is directly related to oocyte quality, sperm-oocyte interactions, and early implantation [212]. Human FF contains numerous low-molecular-weight (LMW) metabolites that serve as direct or indirect regulators of redox balance and antioxidant activity [213]. In vivo, ROS scavengers present in follicular and tubal fluids protect oocytes and embryos from OS [32] (Figure 3). In addition, several antioxidant enzymes, including superoxide dismutase (SOD), catalase (CAT), and glutathione peroxidase (GPx), play key roles in reducing peroxidative damage [214]. These antioxidant systems are critical for maintaining viable and developmentally competent oocytes and embryos [215,216].

Within the ovarian environment, an imbalance in OS adversely affects the development of oocyte [213], embryo [217], and pregnancy outcome [218]. Elevated levels of ROS in the tubal fluid and peritoneal cavity can impair fertilization and early embryonic development [219]. However, some studies report that ROS and lipid peroxidation are not consistently associated with oocyte maturation [218].

Interestingly, the concentration of ROS in FF has been found to be lower than in other culture media [220]. Moreover, women who achieved pregnancy through IVF showed significantly higher FF ROS levels compared with non-pregnant patients [221], suggesting that a certain threshold level of oxidative activity in FF may be necessary for successful conception in IVF cycles [222]. A ROS concentration exceeding approximately 170 cps/µL in FF has been reported as a cohort-specific cutoff above which viable embryo formation becomes less favorable. This cutoff was initially determined in women with tubal infertility and was later examined in patients with endometriosis and polycystic ovarian syndrome (PCOS), showing correlations with fertilization and pregnancy rates [223].

The capacity of oocytes to undergo fertilization and establish a successful pregnancy is highly sensitive to changes in maternal physiology. Advanced maternal age and obesity are associated with lower ART success rates and reduced oocyte developmental competence (ODC), largely due to OS. Oocytes retrieved from obese female mice exhibit altered mitochondrial activity [19,224], irregular mitochondrial morphology [224,225], decreased mitochondrial mass [226], and an increased incidence of chromosomal and spindle abnormalities accompanied by elevated concentrations of ROS [227,228,229], all of which contribute to reduced oocyte viability [227].

Maternal smoking further increases susceptibility of embryos to OS. Exposure of fetal and neonatal rats to nicotine has been shown to increase pancreatic GPx and manganese superoxide dismutase protein expression as well as ROS production [230]. In these rodent models, nicotine also decreases pancreatic respiratory chain enzyme activity and causes degranulation of β-cells, along with increased OS in the islets [231]. As a consequence of rat fetal nicotine exposure, β-cell apoptosis occurs along with elevated OS [232], suggesting that such exposure may predispose offspring to metabolic disorders such as diabetes.

In summary, maternal physiological and lifestyles factors establish the baseline redox status of oocyte, directly impacting subsequent embryo viability. This critical link between maternal health and the initial embryonic environment underscores the need for personalized ART approaches. However, the total oxidative load is not determined by maternal factors alone; the paternal contribution is equally vital, as sperm and seminal fluid quality provide the remaining biological determinants of oxidative balance during fertilization.

## 6. Relationship Between ROS and Sperm and Seminal Fluid

Both male and female factors contribute equally to infertility [233,234]. A substantial body of research has examined the association between ROS and human male infertility [20]. Many studies have focused on OS-related mechanisms underlying human and murine sperm damage [4,235,236]. Under physiological conditions, a controlled level of endogenous ROS generated by human spermatozoa plays an essential role in initiating capacitation and the acrosome reaction, thereby enabling fertilization [4,237]. ROS also act as intracellular signaling regulators; in human sperm, they promote tyrosine phosphorylation and stimulate cyclic adenosine monophosphate (cAMP) production [233], which supports signal transduction required for functional activation. In addition, ROS enhance sperm-oocyte fusion by increasing sperm binding affinity to the oocyte zona pellucida [233]. One study demonstrated that exposure of human spermatozoa to low concentrations of H_2_O_2_ stimulated sperm capacitation and hyperactivation, triggering the acrosome reaction and facilitating oocyte fusion [238].

However, seminal plasma provides protection against such ROS through its diverse antioxidant systems [236,239], including enzymatic antioxidants (SOD, CAT) [240,241] and non-enzymatic antioxidants (ascorbate, GSH, taurine, hypotaurine) [242], which function as free radical scavengers [236]. High ROS levels in human seminal fluid are known to be detrimental to spermatozoa [243]. Because the mammalian sperm plasma membrane is rich in polyunsaturated fatty acids and lacks robust repair mechanisms, it is highly vulnerable to OS [244]. Consequently, elevated seminal ROS levels impair sperm fertilizing ability by inducing DNA fragmentation and apoptosis [245]. Disturbances in seminal fluid redox homeostasis can therefore contribute to male infertility [246,247]. Infertile men exhibit altered systemic and seminal redox profiles, including increased plasma lipid peroxidation and decreased total antioxidant capacity (TAC), particularly in oligoasthenozoospermic individuals [248]. The presence of elevated ROS levels in the semen of infertile patients is strongly associated with decreased sperm variables in men diagnosed with male-factor infertility. Moreover, increased sperm damage caused by ROS shows a positive correlation with higher expression of cytochrome c and the activation of caspases 9 and 3, indicating that excessive OS promotes apoptotic pathways in sperm cells [249].

A previous study proposed an upper critical limit (UCL) for ROS in semen samples from normozoospermic men, beyond which fertilization potential declines. The UCL was determined to be 0.075–0.1 × 10^6^ counted per minute (cpm) per 10 million spermatozoa, representing the threshold associated with normal semen quality [250].

Overall, male factors account for approximately 50% of infertility cases [251]. Research indicates that ROS could be a factor in 30–80% of infertile men [252]. One study using a murine model found that mouse 4-cell embryos derived from conceptions involving paternal obesity have reduced MMP, with MMP driving embryonic energy production through OXPHOS [253]. This resulted in significant delays in mouse preimplantation embryo development. Another study in mice showed that paternal obesity at conception was linked to overexpression of the tumor suppressor *Samd4* in the blastocyst, whose transcription has previously been shown to be strongly associated with OS and ROS concentrations [254]. These findings demonstrate that mammalian preimplantation metabolism, OS, and subsequent embryo quality in vitro can be altered by the father’s lifestyle. However, human and murine sperm have a limited capacity to defend against OS and are susceptible to oxidative DNA damage [255]. DNA damage alters protein synthesis and mitochondrial biogenesis because it damages DNA at both mitochondrial and nuclear levels in spermatozoa, resulting in impaired embryonic development and/or increased morbidity in the offspring [256].

Although physiological levels of ROS are necessary for sperm function, the transition to in vitro environment often leads to excessive OS that compromises paternal DNA integrity and embryo viability. Given the significant impact of ROS on gamete and embryo quality, the development of precise and reliable methodologies for quantifying oxidative status is essential for both research and clinical diagnostic in ART. The following section summarizes the principal methodologies currently employed to measure ROS in reproductive samples.

## 7. Techniques for Measuring ROS

To date, various methods have been developed to measure ROS either directly or indirectly in human and mammalian reproductive samples such as oocytes, embryos, and sperm. Common techniques for direct ROS measurement include fluorescence-based assays, chemiluminescence, electron spin resonance (ESR), and nitro blue tetrazolium reduction (Table 2). In contrast, indirect approaches include TAC, the Endtz test, lipid peroxidation assays, DNA damage assessments, and assays for antioxidant enzyme activity. The practical application of these detections methods is typically divided between research and clinical utilization. Research oriented techniques, such as fluorescence microscopy and ESR, offer high specificity for identifying specific ROS and their intracellular locations but require expensive instrumentation and specialized training. Clinically, the focus shifts toward standardized, rapid and easy to assay. For example, the male infertility oxidative system (MiOXSYS) and luminal-based chemiluminescence methods have been validated for clinical semen analysis.

Among the available techniques, fluorescence-based assays using dichloro-dihydro-fluorescein diacetate (DCFH-DA) are widely used in research settings. DCFH-DA is a cell-permeable reagent for quantifying intracellular ROS in mammalian oocytes and embryos. Non-fluorescent DCFH-DA is hydrolyzed by intracellular esterases to produce non-fluorescent DCFH. Subsequent oxidation of DCFH by ROS converts the molecule into dichlorofluorescein (DCF), which emits green fluorescence. This fluorescence can be measured using a spectrophotometer at 485 nm excitation and 525 nm emission. Fluorescence intensity can be analyzed using ImageJ (version 1.53, National Institutes of Health, Bethesda, MD, USA) software. Studies have used the DCFH-DA probe to quantify ROS levels in embryos [167,257,258]. Typically, embryos were incubated with 10 µM DCFH-DA at 37 °C for 30 min, rinsed with PBS, and analyzed using a fluorescence spectrophotometer at 488 nm excitation and 525 nm emission [163,167]. Additionally, the chloromethyl derivative of H_2_DCFDA (CM-H_2_DCFDA) exhibits better retention in live cells than the conventional H_2_DCFDA probe. This fluorescent probe has been used to measure ROS levels generated by IVF culture media and sheep sperm [259]. As an alternative to DCFH-DA, a cell-permeable and specific green fluorescent probe for H_2_O_2_, Peroxyfluor-1 (an aryl boronate probe), has been used to detect intracellular H_2_O_2_ in mouse pronuclear oocytes [143].

For human semen analysis, luminol chemiluminescence, MiOXSYS, and OxiSperm are commonly used commercial assays. Luminol and MiOXSYS are included in the advanced examination section of the 6th edition of the WHO Manual for Human Semen Examination [260]. In the luminol-dependent chemiluminescence method, a sensitive luminescent probe reacts with ROS (e.g., H_2_O_2_, O_2_^•–^, HO^•^) in whole human semen, enabling measurement of both intracellular and extracellular ROS using a luminometer [261]. The luminometer quantifies light intensity in two modes: (i) photon counting, expressed as relative light units (RLU), and (ii) electric current, expressed as counted photons per minute (cpm) or mV/s [262]. Several studies have attempted to define reference ranges for luminol chemiluminescence for diagnosing male infertility. Research has also explored the association between embryo quality and ROS levels in spent embryo culture media. One study quantified ROS levels in culture media using luminol (5-amino-2,3-dihydro-1,4-phthalazinedione) by chemiluminescence [127]. Similarly, another study used a thermochemiluminescence assay to examine the correlation between human embryo quality and ROS levels in spent culture media [263].

The MiOXSYS method is widely used to determine static oxidation–reduction potential (sORP) in human semen as well as in sperm preparation media, freezing media, and embryo culture medium [264,265]. This system detects electron transfer between oxidants and reductants and provides highly sensitive measurements within 5 min using 30 µL of fresh or frozen human semen [266]. A study by Agarwal et al. reported low specificity and positive predictive values (40.6% and 66.6%, respectively) for fertile men when assessed based on normal semen parameters, but much higher sensitivity and positive predictive values (98.1% and 94.7%, respectively) for infertile men [267]. They also found that human sperm concentration, motility, and morphology negatively correlated with sORP levels, indicating the need for correction based on sperm count. A value of 1.36 mV/10^6^ sperm/mL was proposed as the threshold discriminating fertile from infertile human males [267].

OxiSperm is another assay used to measure excess O_2_^•–^ in sperm, seminal plasma, and whole semen. The assay uses nitro blue tetrazolium (NBT), which is reduced from its yellow form to insoluble blue formazan crystals in the presence of O_2_^•–^ [268]. Several large cohort studies have evaluated the effectiveness of OxiSperm in diagnosing human male infertility, reporting medium to high ROS levels in 31–76% of infertile participants [269,270,271].

In summary, advances in ROS detection technologies have substantially improved the assessment of OS in human and animal reproductive biology; however, harmonization of methodologies and standardized thresholds remain essential for translating experimental findings into clinical practice.

**Table 2:** Analytical methods for detecting ROS in reproductive samples.

Method ^a^	Probe	Sample	Detected ROS	Advantages	Limitations	Ref
Fluorescence-based method (Flow cytometry, fluorescence microscope, micro-plate reader)	DCFH-DA (2′,7′-Dichlorodihydrofluorescein diacetate)	-Sperm (human) -Embryo (mouse, bovine, porcine) -Oocyte (mouse, bovine)	-Intracellular H_2_O_2_ -Intracellular ROS	-Small sample -Can measure multiple markers simultaneously -High specificity	-Different permeabilities to the different types of cells -No clear correlation between the fluorescence intensity and any free radical	[7,116,167,257,258,272,273,274,275,276]
	Dihydroethidium (DHE)	-Sperm (human)	-Intracellular O_2_^•–^	-Cell permeable -Alternative for qualitative measurement of ROS	-Alone is insufficient for O_2_^•–^ quantification	[272,273,274]
	CM-H2DCFDA (5-(and-6-) chloromethyl-2′,7′-dicholorodihydorfluorescence diacetate)	-Embryo (mouse) -Culture media	-Intracellular ROS	-Highly sensitive -Better retention in live cell	-Limited dynamic range	[259,277,278]
	Peroxyfluor 1	-Embryo (mouse)	-Intracellular H_2_O_2_	-Cell permeable -High specificity for H_2_O_2_	-May not penetrate all cellular compartment	[143]
	Amplex Red (N-acetyl-3,7-dihydroxy phenoxazine)	-Sperm (bovine)	-H_2_O_2_	-Sensitive and specific to H_2_O_2_ (with a limit of detection ~5 pmol H_2_O_2_) -Measure extracellular H_2_O_2_	-Slow affinity to cell membrane inability to measure intracellular H_2_O_2_ precisely	[279,280,281]
Chemiluminescence (Luminometer, plate reader)	Luminol (5-Amino-2,3-dihydro-1,4-phthalazinedione)	-Culture media -Follicular fluid (human) -Semen (human)	-Both intra and extracellular ROS (especially O_2_^•–^, H_2_O_2_, HO^•^)	-Robust, sensitive, and specific method -Easy to measure -Readily available	-Large volume sample (~400 µL) -Inability to detect specific ROS -Interference of metal ions in culture media -Light and temperature sensitive -No defined references range	[22,127,217,223,273,282,283,284]
	Lucigenin (bis-*N*-methylacridinium nitrate)	-Sperm (human)	-Extracellular ROS, especially O_2_^•–^, HO^•^	-Highly sensitive to O_2_^•–^	-Lower sensitivity compared to luminol -Limited detection range	[273]
Colorimetric method (Spectrophotometry, bright-field microscopy)	Nitroblue Tetrazolium (NBT), OxiSperm	-Oocyte (ovine) -Cumulus cell (ovine) -Embryo (ovine) -Semen (human)	-Intracellular ROS	-Small sample volume (~30–50 µL) -Highly sensitive -Rapid result (~15 min)	-Low assay precision -Only measure one ROS radical -Subjective analysis	[285,286,287,288]
Electrochemical method	MiOXSYS sensor	-Semen (human)	-Measure sORP (static oxidation-reduction potential)	-Rapid results (2–5 min) -Fast and easy to use -Small sample volume (30 µL) -Highly sensitive -Can be used on fresh or frozen samples	-Temperature sensitive -Cannot differentiate the specific ROS	[267,289,290]
Electron spin resonance (ESR)/ electron paramagnetic resonance (EPR) spectroscopy	-DMPO (5,5-dimethyl1-1-pyrroline-N-oxide) -PBN (N-*tert-*butyl phenyl nitrone)	-Semen (bovine) -Sperm (ovine)	-HO^•^ -O_2_^•–^	-Quantitative analysis of free radicals, and kinetics analysis -Good for high level of ROS production	-If a free radical combines with other molecules, the spin adduct is not formed and cannot be identified by ESR	[279,291,292]

^a^ Note: Fluorescence-based assays, colorimetric methods and ESR are primarily used in research to study intracellular ROS levels with high specificity. Conversely, MiOXSYS and luminol-based methods have high clinical applications.

## 8. Conclusions

ROS are indispensable molecules in normal physiology, playing a dual role in reproductive biology. They are essential for fertilization and embryo development yet become harmful when present in excess. In IVF, gametes and embryos are exposed to elevated ROS levels from external factors such as temperature fluctuations, light exposure, and varying culture conditions, leading to OS that impairs embryo quality and reduces success rates. Although numerous studies have attempted to define safe ROS ranges, establishing an exact threshold remains challenging for both embryos and culture media. The success of IVF cannot be attributed solely to an optimal culture environment; it also depends on maternal and paternal biological factors as well as lifestyle choices. Recently, considerable attention has focused on the use of antioxidants both in vitro and in vivo in ART and reproductive medicine. However, not all antioxidants are beneficial for embryos, and their use alone cannot guarantee improved outcomes. The choice of antioxidant compounds, as well as every procedural step and culture condition, must therefore be carefully evaluated. IVF laboratories should maintain low oxygen culture conditions with precise temperature and pH control, routine redox monitoring of culture systems, evidence-based antioxidant use, and patient-specific oxidative profiling to optimize embryo competence and clinical success. This review provides a fundamental understanding of ROS sources in IVF and offers essential insights to improve ART outcomes and enhance the likelihood of successful embryo development.

## Figures and Tables

**Table 1:** Classification of ROS.

Free Radical ROS	Non-Radical ROS
Superoxide (O_2_^•–^)	Singlet oxygen (^1^O_2_)
Hydroxyl (HO^•^)	Ozone (O_3_)
Peroxyl (RO_2_^•^)	Hydrogen peroxide (H_2_O_2_)
Alkoxy (RO^•^)	Hydroperoxides (ROOH)
Hydroperoxyl (HO_2_^•^)	Hypochlorous acid (HOCl)

## Data Availability

All data supporting the findings of this study are available from the corresponding author upon reasonable request.

## Abbreviations

The following abbreviations are used in this manuscript:

ROS	Reactive oxygen species
IVF	In vitro fertilization
ART	Assisted reproductive technologies
OS	Oxidative stress
ATP	Adenosine triphosphate
OXPHOS	Oxidative phosphorylation
NADPH oxidase	Nicotinamide adenine dinucleotide phosphate oxidase
GSH	Glutathione
pH_e_	external pH
pH_i_	internal pH
FF	Follicular fluid
SOD	Superoxide dismutase
CAT	Catalase
GPx	Glutathione peroxidase
MMP	Mitochondrial membrane potential
TAC	Total antioxidant capacity
DCFH-DA	Dichloro-dihydro-fluorescein diacetate
DCFH	Dichloro-dihydro-fluorescein
DCF	Dichlorofluorescein
MiOXSYS	Male infertility oxidative system
